# Using social media platforms to prepare for examinations post Covid-19: The case of saudi university EFL learners

**DOI:** 10.1016/j.heliyon.2023.e21320

**Published:** 2023-10-21

**Authors:** Abdullah Al Fraidan, Ebtisam Mohammed Al-Harazi

**Affiliations:** English Language Department, College of Arts, King Faisal University, Al Ahsa, Saudi Arabia

**Keywords:** Mobile-assisted language learning, Covid-19, Social media platforms, Exam preparation, Language assessment

## Abstract

Mobile-Assisted Language Learning (MALL) applications have been increasingly used by learners and instructors after the government imposed social and educational restrictions in Saudi Arabia for almost two years due to the Covid-19 pandemic. This research investigates Saudi EFL university learners’ use of MALL applications, specifically Social Media Platform (SMP) applications, in their preparation for achievement exams post the Covid-19 pandemic social and educational restrictions. A questionnaire consisting of questions, as well as a range of open-ended question was designed and administered to 125 English major female learners who were enrolled at King Faisal University (KFU) in two different educational modes viz., regular (66) and distance (59) participation. Seven SMP applications – WhatsApp, YouTube, Telegram, Twitter, Instagram, Snapchat and Facebook – were used in this study. Data were analyzed quantitatively using the MANOVA test in SPSS, and qualitatively. The results have shown that both Regular Learners (RLs) and Distance Learners (DLs) used SMPs to prepare for their exams. The data analysis revealed that there were significant differences between RLs and DLs in their frequency of use of SMPs in preparation for exams. WhatsApp was the most frequently used SMP by RLs, whereas Telegram was the most frequently used SMP by DLs. Facebook was the SMP least frequently used by both groups. There were also statistically significant differences in favor of RLs over DLs in the frequency of use of SMPs in preparation for exams due to the Covid-19 pandemic Most participants reported that SMPs have a positive effect on their academic achievements. During the pandemic this was the main reason for continuing to use SMPs. Other reasons that emerged from the qualitative analysis of the open-ended responses included educational, technical and cognition reinforcement. The study results suggested implications for learners, instructors and educational policy makers.

## 1 Introduction

The rapid adoption of Mobile-Assisted Language Learning (MALL) applications has led to their integration into the educational processes. These MALL applications have been increasingly used by learners and instructors after social and educational restrictions were imposed in Saudi Arabia for almost two years due to Covid-19. The Saudi government suspended schools for more than one year and moved all students to attend schools through an online local platform called My School (Madrasati, in Arabic) while universities used the Blackboard platform. Mid-2021, the Saudi government eased these restrictions by allowing different groups of students to attend regular schools and other groups to attend by distance mode in the online platform. employed.

Prior to Covid-19, Saudi learners and instructors used different MALL applications mostly for social purposes with little educational usage (see for example [1]; Aljumah, 2012 [2]; Parkes & Adlington, 2017). But that usage was only an optional and supplementary resource in a limited number of courses at the university level. With the greater application of technology during the Covid-19 pandemic, learners started to use different MALL applications, especially SMPs, to share information, announcements and learning materials for their online classes and exams. When students returned to normal classes after eighteen months, a question arose as to whether Saudi EFL university regular and distance learners should continue to use SMPs to prepare for their achievement exams. The focus of the present study was to observe what SMPs the learners continued to use post-Covid 19 pandemic and whether using these platforms affected students' learning positively or negatively. The results of this study were expected to provide instructors with the knowledge of the effects of these platforms on students' performances in examinations. Moreover, the study has shed light on the platforms most frequently used by learners for their exam preparations. By recognizing these platforms, instructors can better integrate them into their learning environment thus enhancing the students’ learning and their grade achievements.

### 1.1 The importance of testing and the preparation for tests

Factors that affect students' success in their exams vary. They include the learning environment, teacher, quality of education, teaching methodology, teaching and learning tools, students' mates, personal study habits, motivation and test-taking anxiety [3]. Bıçak argues that in addition to all these factors, effective preparation for exams with a selection of appropriate test-taking strategies is a key step that can enhance students’ success. Test preparation strategies are attracting considerable interest due to their effects on test scores. It is well-known that test preparation is important to the test-takers, the test developers, the test sponsors and the score users [4].

Some researchers use the term “test preparation” interchangeably with test-taking strategies. However, there is a difference between the two terms: the latter is used to refer to the strategies that students use while tackling tests, and the former is used to refer to the strategies that students use beforehand to prepare for the test. Researchers, who focus on special test preparation–coaching, consider test-taking strategies as part of the test preparation. The focus of this study is on the latter, namely the test preparation.

### 1.2 Importance of social media platform applications research

Social networking sites have revolutionized and changed the ways in which people communicate and exchange information [5]. Ubiquity is a special facility in the new media technology that is taken for granted by today's generation. MALL applications, as examples of these technologies, have a positive role in enhancing different language skills in the context of foreign language learning regardless of these skills being oral or written. The essential affordances of such platforms when they are downloaded to mobiles are that of permanency, immediacy, accessibility and interactivity [6]. Additionally, MALL applications are the most commonly-used tools by students [7]. These applications facilitate students' interaction with one another and with their instructors as well. Social Media Platforms are the most popular MALL applications widely used by EFL learners in Saudi Arabia. In these platforms, clusters can be created for students to form study groups for discussion, interaction, seeking help to learn any language skill and discussing module material [5,8]. Although students and educators are aware of the positive effects that SMPs can bring to the education sector, there is a need, especially in Saudi Arabia, for more investigation into the benefits of integrating these SMPs into education [9].

### 1.3 The educational system in Saudi Arabia

The vision of the Ministry of Education in Saudi Arabia is to create a globally competitive knowledge-based community and to combat illiteracy.

English language is integrated in the government and private schools’ curricula. English is a compulsory subject from the first grade in the elementary level.

There are 27 government universities, 10 private universities and 42 autonomous colleges at the tertiary level in Saudi Arabia. All institutions award a variety of education qualifications in many different fields. Universities also award different levels of degrees: Bachelor, Master and Doctoral degrees. KSA. provides equal education to both genders at all levels. Several attempts have been made to improve the education process and its outputs. One of these was the implementation of online teaching tools and platforms, such as Blackboard.

King Faisal University (KFU), the study context, was one of the first universities in Saudi Arabia to implement distance and online programs and platforms. It was a pioneer university not only locally but also at a regional level.

### 1.4 The motivation behind the present study

Saudi Arabia faced the need for fundamental change in the educational process in order to overcome the effects of Covid-19 by the accommodation of technology, relying on it for strong technological infrastructure. Students started attending their classes online through the My School platform and 12 free educational satellite TV Channels for K-12 grades called EIN (Eye in English) with expert instructors explaining all the K-12 grade courses on these channels. This fundamental change caused students to rely more on using SMPs in their learning [10]. SMPs recently have become very useful tools to improve language proficiency with no restrictions of time and place. However, the existing body of research on the use of SMPs has only focused on the process of teaching or learning English [[11], [12], [13], [14]]. Most studies in Saudi Arabia focused on how social media raised the health awareness of Covid-19, or shared information about it. No study looked at how students used SPMs to prepare for their exams. This study attempts to fill this knowledge gap.

KFU provides its undergraduate program and courses in two modes: regular mode with traditional teaching in a classroom, and distance mode using online tools such as Blackboard for more than 140,000 students across the country. The distance-mode students are familiar with educational technology tools since most of their studies are undertaken online and they are heavily reliant on SMPs to share learning materials, samples of past exams and important announcements.

With the onset of the Covid-19 pandemic, all university students were shifted to distance mode learning as the university had the required infrastructure and substantial existing experience in online education. The researchers noticed that the regular students used SMPs to communicate among themselves. After all educational restrictions were lifted, the researchers wanted to establish whether the students, especially the regular students, continued using SMPs in their learning and exam preparation. The current study aims to contribute new evidence to the field of applied linguistics by exploring the use of SMPs to prepare for achievement exams with the focus on Saudi Arabian female university students majoring in English using the two modes of teaching (regular and distance). The associated research questions are as follows.

Q1 Did Saudi Arabian EFL university learners continue to use SMPs to prepare for achievement exams post the Covid-19 pandemic?

Q2 What were the SMPs most frequently used by Saudi Arabian university EFL learners to prepare for their achievement exams?

Q3 Was there a difference between regular and distance Saudi Arabian university EFL learners in their use of Social Media Platforms in exam preparation?

## 2 Literature review

### 2.1 Test preparation

the extant literature, reveals there are two main approaches to test preparation. The first approach has been termed by many researchers as coaching. It basically looks at test preparation courses mostly for high stakes exams such as IELTS, TOFEL, and SAT [4,15,16]. According to the dictionary of the National Council on Measurement in Education (NCME), coaching refers to “planned, short-term activities for prospective test-takers that are intended to maximize the scores of those individuals on an upcoming test”. It expands the definition by describing the activities as those which “may include new learning, practice with previous learning, and using test-taking strategies”. Chung- Herrera et al. (2009) add test-witness to the previously mentioned activities. The second approach, looks at the students’ behaviors when they study and is called a self-initiated test preparation [17]. have defined this approach as “activities that involve studying or preparing for the content of the test outside of any formal preparation program” (p. 1210). The two approaches have significant effects on the performance of students in tests [17].

Test preparation, in some previous definitions of study habits is considered a part of study habits and skills that form the learning procedures. For example [18], defines study habits as the activities that learners employ when dealing with the subject of study. He elaborates the concept by describing study habits as the procedures that learners can acquire, formulate and cultivate. Furthermore [19], define study habits or skills as compulsory activities that are utilized by learners for the completion of the academic task demands and for tests preparation [20]. see preparation for exams as one activity of study habits [21]. considers any activity that assists learners to learn, train or prepare for tests as test preparation. In the NCME, test preparation is defined as “a number of activities in which a prospective test-taker might participate, primarily for the purpose of optimizing their scores on an upcoming test”.

Another type of test preparation is studied by Ref. [21] who believes that test preparation has many forms depending on the test formats. According to him, test preparation concerns what instructors see as essential skills to pass a test. He studies communicative test preparation which was utilized to prepare for communicative language testing. This type of preparation is grounded n Canale & Swain's (1980) communicative competence. Another belief in test preparation is held by Refs. [22,23] who argue that test preparation practices are associated with language learning and the use of appropriate strategies [23]. states that learning and study strategies are test preparation activities that affect test scores. It is her belief that test preparation activities intend to provide test-takers with information of a particular field in which they will be tested [24]. uses the term Test Preparation as “associated with activities inside and outside classes that help students be ready for exams. These activities usually focus on familiarizing students with test formats, acquainting them with instructions included n the test and also providing them training on time management” (p. 5).

Accordingly, it is the second view of test preparation activities, the informal, that is considered in this study. This notion overlaps with other concepts such as study habits, learning strategies, study strategies and self-initiated test preparation [22].

Although many students and educators believe that 10.13039/100009287MALL applications can support and foster learning, education, language teaching and learning, their educational applications are still low [25]. It is apparent from what has been presented that the current generation, known as being “digital savvy” [26,27], favor the use of SPMs not only for socilization but also for educational purposes. Of course, Saudi students are no exception. Even though there are several studies about MALL applications which were conducted in Saudi Arabia, there is still a need for more investigation, specifically in Saudi higher education [28]. Given this scenario, the current study aims to contribute to the growing area of research by exploring the role of SMPs being used in exam preparation among Saudi EFL university learners in regular and distance modes.

To many scholars, MALL applications are facilitators for learning processes where they play an effective role in facilitating learning through collaboration, communication, motivation [29,30] and information sharing [26,31] [13,32,33].

Theories that can be applied in MALL applications and reviewed in the literature are mostly induced from early reported theories of learning in which collaboration and social interactions are the main elements. As for the theories that could underpin this study, different theories branch out. Among those different theories: Social constructivism and connectivism were selected.

Social constructivism theory underpins much research that is related to the use of Web 2.0 tools and methods [34]. Social constructivism views learning as a social process and learning is thus socially developed [35]. The theory is rooted in Ref. [36] early theory which stressed that learning occurred through social interaction [36]. also articulated the notion of the “more capable/knowledgeable other”, that could be a teacher, another student or any other capable person. In the current study, learners can interact via SMPs with ‘a more capable other’ such as professors and more successful students who can assist less successful students. Another essential conceptualization for Web 2.0 and collaborative work that is usually related to constructivism and social constructivism, is the concept of connectivism [34]. Connectivism is a model that considers learning as a process that can be facilitated through maintaining connections and interactions between people. The network is an integral concept in the theory of connectivism.

Moreover, SPM technologies are the infrastructure in distance mode learning [37]. Thus, the idea of engaging learners in collaborative learning, where learners work together, share knowledge, exchange ideas and support each other, fosters the process of learning and assists in knowledge construction [38]. Based on social constructivism theory, social interaction can be fulfilled in distance mode as a key factor in creating knowledge. The interactions of people in a web-based learning environment using “SMPs in the case of this study”, assist to create, sustain, and support associated learning processes. In this regard, collaborative online discussions within SMPs stand as worthwhile examples. The result of the current study would contribute to the knowledge of the two relevant theories that could underpin this study.

### 2.2 The use of social media in learning

[[39], [40], [41]] acknowledge that collaborative learning is an advantage of using SMPs [9,33,42]. note that one of the great aspects of using MALL applications for a learning purpose is creating an endless connection between students and their instructors.

[43] found that among all other platforms (i.e., YouTube, blogs, forums, wikis, Facebook, Instagram, LinkedIn, Twitter, Weibo, WeChat and WhatsApp), YouTube was the most widely used for English Language Learning (ELL), followed by Facebook, wikis and WhatsApp. In her study, YouTube and wikis were considered official platforms as they are controlled by the institution.

Different studies in the Saudi context proved the effectiveness of using various SMPs to enhance learning (see Refs. [12,39,44,45]. However, none of the studies mentioned the use of these SMPs as a tool for test preparation. Other studies explored the perception of students in regard to the use of SMPs (see Refs. [5,46].

Students also use SMPs for other learning purposes. A study by Ref. [47] explored the effectiveness of using WhatsApp to improve students' learning and exam performance. Data were collected from 92 students at a university in Istanbul. The students were allocated to a control group (32 participants) and an experimental group (60 students). The control group studied using traditional learning methods while the experimental group studied using WhatsApp by messaging, asking questions and receiving answers. The results showed that the participants used WhatsApp to study because it encouraged collaborative work. The researcher found that students’ use of WhatsApp to study influenced their achievement positively and that small WhatsApp groups tended to be more beneficial than larger ones. Other studies found that SMPs could increase self-determination, motivation, autonomy, competence and relatedness [26,[48], [49], [50], [51]].

[52] used a quantatiative study to explore instructors’ use of SMPs to teach EFL. Seventy-five male and female instructors were chosen randomly from two Saudi tertiary institutions. A questionnaire was used to collect data. It was found that instructors strongly believed that social media tools played a significant pedagogical role in EFL classes. However, the partcipants reported that SMPs may have a double-edged sword effect, distracting learners from the intended learning. They concluded there is a need for other studies that can suggest the development of best practices in the integration of SMP tools into the process of English Lnaguage learning and teaching. This is the objective of the current research.

[28]; explored the use of SMPs by EFL learners at King Saud University, based on 1479 students (934 male and 545 female) from the Preparatory and First years. Students were asked to state their reasons for using SMPs. Most respondents reported that they primarily used SMPs to communicate with their classmates and their instructors about the topic related to their study. The study revealed that Saudi higher education students regard SMPs as a learning tool to meet their educational needs rather than a platform for socialization. The current study attempts to complement this study by demonstrating that test prepration could be another important reason for using SMPs.

The investigation of SMPs in the domain of language learning and teaching continues as the focus of many researchers in KSA [11]. investigated the use of WhatsApp in EFL teaching at a university in Saudi Arabia. The study utilized focus group interviews, with participants drawn from EFL preparatory year students and their faculty members. Thematic analysis was used to analyze the obtained data. The results showed that both students and faculty members who participated in the study had a positive attitude towards using WhatsApp in EFL teaching. Such attitudes of the learners are similar to those of students who participated in the work of [1]; giving us a clearer view of the importance of SMPs as a learning facilitator. In this regard, the [11] findings showed that students mainly use WhatsApp for information exchange, language learning support and language practice. The analysis of the interviews also revealed that using WhatsApp can support three main instructional strategies: teacher-directed learning, peer learning and independence in learning. Three major themes emerged from the analysis of both students and faculty members, namely the affordances of WhatsApp, affective outcomes of using WhatsApp and learning English using WhatsApp. Each major theme was divided into sub-themes as follows.

1. Affordances of WhatsApp: personalization, immediacy, flexibility and interactivity.
2. Affective outcomes of using WhatsApp: confidence, trust and concern.
3. Learning English using WhatsApp: informal, accessible and the teacher's role as a facilitator.

This study added to the literature in the use of one of the SMPs in EFL learning [11]. shares a similar context to the present study. Such work provides us with useful information about the nature of the students and their attitudes toward using one of the targeted SMPs, namely WhatsApp, which was recently proven to increase learners’ autonomy (Aalmer & Al Khateeb, 2023). Learners autonomy is one of the successful steps towards good test preparation [53].

Overall, the literature on MALL applications either focuses on one of the SMPs (e.g., WhatsApp, and Telegram) or on all SMPs in general. Previously reviewed studies showed that most of the studies conducted on SMPs in relation to the educational field as well as language learning, investigated areas such as collaborative learning, learners' interactions, peer-feedback, social relations and communication. Similarly, those areas are related to what has been identified by many researchers in language learning strategies (LLS) as Social Strategies. For example [54], added social/affective strategies to the categorization of LLS, including interaction and collaboration with other students [55]. includes seeking help while handling tasks, asking for an explanation to a confusing point, and cooperating and interacting with others [56]. involves students’ choice of actions to interact with other learners. Recently SMPs have been linked to lowering test anxiety [57] but were not proven to be used by university students to prepare for their course tests, this being the main focus of the present research.

## 3 Methods

The study used a triangular method for collecting data through an online survey, individuals and focus groups. Both quantitative and qualitative analysis were used for this purpose.

### 3.1 Instruments

Two instruments were used in this study. The first was a questionnaire-based survey developed by the researchers comprising five sections of closed-response questions (e.g., binary Yes/No responses, multiple responses and Likert-type scaling) and open-response questions. Section 1 of the instrument asked the respondents to provide demographic information (e.g., gender). Section 2 was specific to the use of SMPs (Types of SMPs used in exam preparation). Section 3 asked about the usefulness and helpfulness of SMPs. Section 4 focused on the frequency of use of SMPs in exam preparation. Finally, section 5 solicited information related to the students’ perceptions of the effects of SMPs on their learning and exam preparation, and the features that students preferred in the SMPs for exam preparation. Open-ended questions were also included for participants to add more information where applicable in every section. A pilot study was conducted in order to check the validity and reliability of the research survey.

The second instrument comprised semi-structured interviews with 22 participants randomly selected equally from the RLs and DLs groups.

### 3.2 Reliability of the instrument

To test the reliability of the questionnaire, Cronbach's Alpha (α), described by Brown (1997) as sufficient to test reliability, was used by the researchers to assess the pilot study. Table 1 below shows the reliability of the questionnaire.

**Table 1:** Reliability of the questionnaire.

Questions	N Items	Cronbach's Alpha
First	8	.816
Second	13	.915
Questionnaire Reliability	21	.929

It is evident from Table 1 that the Cronbach's alpha for the overall items (21 items measuring 0.929), indicates the high reliability of the questionnaire. The measures for each subscale ranging from 0.915 to 0.816 also indicate the high reliability of the questionnaire's components. According to Ref. [58] an alpha of 0.70 is a sufficient indicator of the instrument's reliability. As the Cronbach's alpha for the pilot exceeds this threshold, the proposed questionnaire is a suitable instrument for use in the main survey.

### 3.3 Validity of the instrument

To determine the validity of the questionnaire's content, the instrument was sent to reviewers who are professionals in the field of applied linguistics (Appendix 2), accompanied by the scope and aims of the study. Considering the reviewer's comments and recommendations, the instrument was revised and modified prior to administration. The final form questionnaire was used in the pilot study as one way to ensure the questionnaire's internal validity. Internal validity was checked using Pearson correlation in the Statistical Product and Service Solutions (SPSS) software. Table 2, Table 3 show the Pearson correlation analysis of the two parts from the third section in the questionnaire, which contain the essential questions.

**Table 2:** Pearson Correlation analysis for the first part of the questionnaire.

Items	Pearson Correlation	Sig. (*p* value)
1	.773^a^	.000
2	.635^a^	.000
3	.730^a^	.000
4	.429^a^	.009
5	.606^a^	.000
6	.738^a^	.000
7	.759^a^	.000
8	.677^a^	.000

a Correlation is significant at the 0.01 level (2-tailed).

**Table 3:** Pearson Correlation analysis for the second part of the questionnaire.

Items	Pearson Correlation	Sig. (*p* value)
1	.822^a^	.000
2	.849^a^	.000
3	.773^a^	.000
4	.680^a^	.000
5	.719^a^	.000
6	.497^a^	.002
7	.695^a^	.000
8	.514^a^	.001
9	.829^a^	.000
10	.716^a^	.000
11	.792^a^	.000
12	.399^b^	.016
13	.995^a^	.000

a Correlation is significant at the 0.01 level (2-tailed).

b Correlation is significant at the 0.05 level.

### 3.4 The subjects

There were 125 participants in the research. The participants were English language major students of two distinct types of enrollments. The first group consisted of 66 female students enrolled in the regular mode of learning and the second group comprised 59 females enrolled in the distance mode of learning. Both groups studied at King Faisal University. The students’ ages ranged between 18 and 30 years. Since the survey was distributed online, the selection was random. The 22 subjects selected for interviews were also randomly selected. The two groups were compared as they shared the same program (a skill-based English program), and they took the same type of examinations which were achievement tests. For cultural reasons in Saudi Arabia, the researchers chose only female students especially for accessibility during the personal interview sessions. Gender was not an important variable in this study, which may provide a new venue for future research.

### 3.5 The procedures

The research utilized a written questionnaire as a main instrument for data collection. Prior to designing the questionnaire, related literature was reviewed to gain more insights about the way students use SMPs to foster their learning.

Based on the literature review findings and after setting up the structure of the research questions, the individual items of the questionnaire were formulated by the researchers. The draft questionnaire was given to reviewers for opinions and comments. After amending the questionnaire according to the reviewers' suggestions, the questionnaire was translated into Arabic as the respondents’ first language (L1) to obtain more reliable responses. An expert translator checked the translated version of the questionnaire following which it was approved by a translation agency. The electronic link to the survey was sent first to four students in order to check the overall form of the questionnaire and its accessibility. The questionnaire was administered online, and responses were generated randomly. The focus of the study was on female students; thus, all male responses were deleted, resulting 125 valid responses from female students. The data gathering took nearly three weeks, and the subsequent coding and analysis of data took nearly two months. The responses were analyzed both quantitatively and qualitatively. The data from the interviews were analyzed into different thematic codes. The coding was validated by another colleague researcher who is an expert in qualitative analysis. The results from the interviews were used to supplement the findings from the survey, clarify the uses of SMPs, and the reasons for their use. The data were analyzed quantitatively using the MANOVA test in SPSS.

## 4 Results & discussions

This section reports and discusses the results of the analysis and organized in four different sections. The first section answers RQ 1 about the types of SMPs used in exam preparation. The second section answers RQ2 about the most frequently used SMPs in exam preparation. The third section answers RQ3 about the differences between RLs and DLs in their use of SMPs. Finally, the fourth section presents the results of the open-ended questions.

### 4.1 SMPs that RLs and DLs use in test preparation

Since the main aim of this research is to examine the use of SMPs among Saudi EFL students, the first question in the survey required students to indicate whether they used SMPs to prepare for examinations. The first research question answered in this section was.

RQ1 Did Saudi Arabian EFL university learners continue to use SMPs to prepare for achievement exams post the Covid-19 pandemic?

The answer to this question is in the affirmative. This study has identified that Saudi EFL university learners continued using SMPs as their main tools for different learning purposes and one of the ways to prepare for their exams. Another question that was triggered by the first was: What types of SMPs do RLs and DLs use to prepare for exams?

**Fig. 1:**
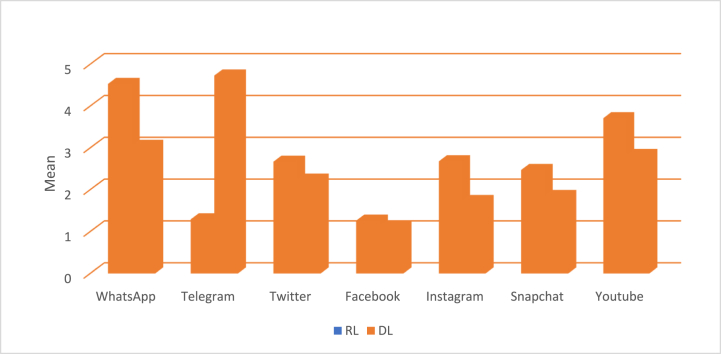
Social Media Platforms: frequency of use by RLs and DLs.

As shown in Fig. 1, the present study revealed that Saudi EFL university learners chose from an array of seven different SMPs to prepare for their exams. An interesting finding was that the SMPs used by RLs in exam preparation differed from those used by the DLs. The results showed that RLs used the entire array of seven different SMPs, though they had a marked preference for WhatsApp and YouTube, and a much lesser inclination to use Twitter, Instagram, Telegram, Facebook and Snapchat, in that declining order. DLs, on the other hand had a marked preference for Telegram to prepare for their exams, and a much lesser inclination to use WhatsApp, YouTube, Twitter and Snapchat in that declining order.

RQ2 What were the Social Media Platforms most frequently used by Saudi Arabian university EFL learners to prepare for their achievement exams?

In response to this research question, the following results were obtained.

a) The most frequently used SMPs by RLs to prepare for exams was WhatsApp followed by YouTube while the least frequently used SMP by RLs was Facebook.
b) The most frequently used SMPs by DLs to prepare for exams was Telegram while the least frequently used SMP used by DLs was Facebook.
c) Table 4 shows the multivariate MANOVA result of the difference in the use of the various mobile applications based on the enrollment type (regular vs. distance), which was non-significant (F (2,10) = 3.718), p = 0.062, Wilk's Λ = 0.573, partial η2 = 0.427). Table 4 also shows the frequency with which the seven SMPs were used for exam preparation by RLs, ranked from 1 to 7 and portraying mean frequency scores ranging from 4.53 (WhatsApp) to 1.26 (Facebook). The mean scores were accumulated for each SMP across the five Likert scale scores by assigning values from Frequently (=5) through to a value for Never (=1), multiplying the relevant frequency by the Likert score assigned. That is, for DLs and WhatsApp multiplying as follows: [((46*5) + (11*4) + (7*3) + (2*2) + (0*1))/66 = 4.53].

**Table 4:** MANOVA results: Comparison of DLs and RLs frequency of use of SMPs.

Mobile-Assisted Language Learning applications	Type of enrollment	N	Mean	SD	F	P.value
WhatsApp	Regular	66	4.53	0.81	3.946	0.000
	Distance education learners	59	3.05	1.42		
Telegram	Regular	66	1.29	0.55	3.195	0.000
	Distance education learners	59	4.73	0.61		
Twitter	Regular	66	2.67	1.33	2.462	0.061
	Distance education learners	59	2.24	1.26		
Facebook	Regular	66	1.26	0.47	2.232	0.051
	Distance education learners	59	1.12	0.33		
Instagram	Regular	66	2.68	1.51	3.776	0.000
	Distance education learners	59	1.73	1.31		
Snapchat	Regular	66	2.47	1.71	3.541	0.031
	Distance education learners	59	1.85	1.35		
YouTube	Regular	66	3.71	1.15	3.299	0.008
	Distance education learners	59	2.83	1.38		
**Total usage**	Regular	66	2.66	0.78	3.718	0.62
	Distance education learners	59	2.51	0.69
d) Both the means for RLs and DLs were mid-range values. This may probably be explained insofar as both groups preferred to use certain SMPs, frequently and using them for exam preparation over other platforms.
e) While we see that WhatsApp and Telegram receive the highest mean scores (4.53 and 3.05 respectively), one may investigate the other participants who did not report them as important platforms to prepare for their exams. This could be a new venue for future research.

The third research question in this study was an attempt to examine the differences between RLs and DLs in their use of SMPs.

RQ3 Was there a difference between regular and distance Saudi Arabian University EFL learners in their use of Social Media Platforms in exam preparation?

With respect to the frequency of use of each of the SMPs, the findings showed that DLs and RLs did not significantly differ from each other in their frequency of use of Twitter and Facebook. Nevertheless, the two student groups did significantly differ from each other in the frequency of their use of WhatsApp, YouTube, Snapchat and Instagram with RLs reporting higher means for each of the nominated SMPs than DLs. Regarding the frequency of use of Telegram, the findings revealed significant differences between RLs and DLs, where the DLs mean was markedly higher than the mean of RLs.

As Table 4 shows, there were no significant differences between DLs and RLs in their frequency of use of Twitter and Facebook to prepare for exams, where (F = 2.462, p = 0.061) for Twitter and (F = 2.232, p = 0.051) for Facebook. In both cases the p-value is greater than 0.05 and thus not statistically significant.

However, the results of the MANOVA analysis showed significant differences between the two groups in their frequency of use of the other SMPs, i.e., WhatsApp, Instagram, Snapchat and YouTube, where RLs reported a higher mean score than DLs. The univariate MANOVA results, as shown in Table 4, are as follows: WhatsApp, Instagram and YouTube F = 3.946, F = 3.776, F = 3.299 respectively and p = 0.000 for all three. Snapchat was also significant (F = 3.541, p = 0.025). The results of the MANOVA analysis also revealed statistically significant differences between DLs and RLs in their frequency of use of Telegram with DLs reporting a higher mean score (4.73) than RLs (1.29).

It was surprising that the differences in the frequency of use of most of the SMPs between both groups, is more marked for RLs although DLs are more dependent on e-learning than the RLs. These differences can be explained by the influence of the Covid-19 pandemic causing RLs to rely more on SMPs than otherwise might have been the case. This may also be attributed to other variables such as learners’ age, employment and their social status that led them to choose distance education. Most of the DLs reported in interview that they have several social and employment commitments, and thus chose to study in this mode. Here is a translated quote from the interview of one of the DLs:

“I have a job in the morning and study with my children in the afternoon, I have lots of commitments, so I do not have time to look for my own study. I found Telegram students groups with all my needs. So, I do not to waste time looking in WhatsApp or YouTube.”

It can be assumed that it is more convenient for DLs to focus on one resource such as Telegram to access what they need for exam preparation rather than spending time on other SMPs to understand unclear points and seeking answers to their inquiries. A further study with a focus on these variables for DLs is suggested.

The analysis revealed that RLs found that WhatsApp is the most frequently used platform to prepare for exams followed by YouTube, and this finding is consistent with [1,7,59]; and [11] who all found that Saudi learners prefer using WhatsApp and Telegram over other platforms.

Regarding the differences between RLs and DLs, the MANOVA was used to identify the statistical significance of the differences in the students’ use of SMPs in exam preparation based on the type of enrollment. The results are shown in Table 5.

**Table 5:** MANOVA results: A comparison of helpfulness and usefulness between RLs and DLs in using SMPs.

	Regular Learners			Distance Education Learners			F	Sig.
	N	Mean	Std. Deviation	N	Mean	Std. Deviation		
**Helpfulness**	66	4.03	0.61	59	4.27	0.51	3.944	0.015
**Usefulness**	66	3.62	0.60	59	4.17	0.46	6.381	0.000

Regarding the helpfulness of SMPs, we used both quantitative and qualitative methods to analyze this issue from responses to both instruments relating to the same question, namely, how can SMPs help you in exam preparation? The multivariate MANOVA result of the difference in the helpfulness and usefulness of the various mobile applications based on the enrollment type (regular vs. distance) was significant (F (4,34) = 5.612), p = 0.002, Wilk's Λ = 0.622, partial η2 = 0.377). It is clear from Table 5, showing the MANOVA result, that there is a statistically significant difference between RLs and DLs in terms of the extent to which SMPs help students in preparation for exams, with DLs reporting a somewhat higher mean score than RLs in terms of helpfulness, ((F (3,41) = 3.944, p = 0.015, Wilk's Λ = 0.573, partial η2 = 0.427), and for usefulness ((F (5,36) = 3.718, p = 0.000, Wilk's Λ = 0.794, partial η2 = 0.224). By exploring the qualitative data from both the interviews and the open-ended questions in the survey, we found that DLs reported that they are constantly engaged in an online environment, so exam preparation is not entirely linked to SMPs. On the other hand, RLs reported that SMPs are the only online accessible platforms available to them in order to connect with each other or with their instructors. One RL said in the interviews:

“*I got used to online resources while we were in the Covid-19 period, especially SMPs. But now we are back to regular classes. I can’t stop using SMPs for my study or exam preparation. So, still find WhatsApp so helpful and useful.”*

The participants were also asked: How useful are SMPs when preparing for exams? It was found that there is a statistically significant difference between DLs and RLs in terms of the usefulness of SMPs in exam preparation, with DLs reporting a higher mean score than RLs, where p = 0.000 which is less than 0.05. This may be explained by the fact that DLs are more experienced and more familiar with the use of electronic sources. SMPs offer avenues for DLs to interact with each other, offering them a class-like environment. Consequently, using SMPs in exam preparation keeps them focused and bridges the distance gap that exists in the distance education mode of learning. Using SMPs also helps DLs to be exposed to more and different inputs as they do not attend regular classes. This finding supports [60] viewpoint, that the use of two-way communication media is the infrastructure and bones of distance education. It is also consistent with [37] who argue that the use of mobile technologies is an indispensable component in distance education.

Only 4.8 % of participants reported that SMPs may have a negative impact on their learning. This is an exceedingly small proportion especially given that SMPs undoubtedly have their limitations. Distraction, distrust, and time consuming were the reasons that students yielded as disadvantages of using SMPs. These reasons are in line with the findings of [61] who categorized distraction and distrust as the disadvantages of using SMP tools in English for Specific Purposes (ESP) courses.

With respect to the helpfulness of SMPs in exam preparation, the results showed that significant differences exist between the two groups. The mean of the DLs was higher than the mean of RLs. This was probably due to the effect of the mode of study i.e., lack of online resources in the regular mode and the effect of Covid-19 caused RLs to find SMPs more useful and helpful.

Finally, the study also yielded some interesting qualitative results through answers to some of the open-ended questions shown below. Most participants reported that the period of the Covid-19 pandemic was the main reason that enhanced the value of using SMPs, so they continued using SMPs to prepare for exams as it proved to have a positive impact on their learning. Both DLs and RLs reported that using SMPs to prepare for their exams caused them to be more confident and less anxious as they felt they studied together using the same materials. We found this among more than 70 % of the students. One student said:

“*I do not feel lost, I don’t feel I missed anything. With studying with others, you feel so confident and less anxious that you may missed studying something.”*

The following questions were used to obtain the participants’ responses.

• Does using SMPs in exam preparation affect your learning positively or negatively? How?
• Was the Covid-19 era the reason that made you prefer and continue using SMPs to prepare for exams?
• Please add any additional answers regarding how you use SMPs in exam preparation.

Beside reporting the effectiveness of SMPs in enhancing participants' learning, exam preparation and lowering test anxiety, students also reported in the interviews different reasons for using SMPs. These reasons included ease of use, ease of access, availability of materials, preference for collaborative learning, speed of getting a response to enquiries, and enhancing the learners’ autonomy.

## 5 Conclusion

The primary objective of this study was to determine whether, and to what extent, Saudi EFL University learners use SMPs to prepare for their exams. The study investigated the frequency of use of SMPs by both DLs and RLs to prepare for exams, and examined whether differences exist between the two types of EFL learners, RLs and DLs, in their use of SMPs in exam preparation. Seven SMPs were used in this study: WhatsApp, Telegram, YouTube, Twitter, Snapchat, Instagram and Facebook. The study comes at a time when there is a demand in Saudi Arabia for studies that deal with the use of SMPs for learning purposes and how to educate students to take advantage of them [62].

The first section shed light on the importance of testing and test scores, introduced the context, presented the importance of the topic, its personal interests and stated the research questions. The second section reviewed the available literature related to the current work through which it provided insights into the use of SMPs in education and language learning. Section three presented the methodology used in conducting the present study where the main data collection tool was delineated, and its reliability and validity checked. Section four presented the results and discussed them. The findings clearly indicated that both RLs and DLs use SMPs to prepare for exams. The study has also shown that RLs and DLs differ in their use of SMPs in exam preparation and in their frequency of use of these SMPs. The study contributed by pointing out the importance of the two most frequently used SMPs to be integrated in the teaching and learning process. Another significant contribution of this study is that the use of SMPs by Saudi EFL university learners had a positive impact on their learning, exam preparation and lowering test anxiety, hence enhancing their overall exam scores. It also contributed to the field of MALL by presenting different reasons for using seven popular SMPs. The last section summarized the research main aims and findings, drew conclusions about the research questions, and provided some implications of the study and recommendations for future research.

### 5.1 The implications of the results

The aim of the current study was to find out whether Saudi university EFL learners use SMPs to prepare for their achievement exams. The investigation went further to explore the types of SMPs that learners use in their exam preparation, and to ascertain the differences between DLs and RLs in their use in exam preparation. The present study provides theoretical as well as pedagogical implications.

Regarding the theoretical implications, this study will serve as a base for future studies as it proposed a valid and reliable questionnaire, written in both English and Arabic languages that can be applied to other future research that investigates the use of SMPs for exam preparation. This ready-made instrument can save the time and effort of other researchers.

The present study has provided additional evidence with respect to the use of SMPs for educational purposes. It contributes to the recent literature of [63] by providing confirmatory results showing that the use of SMPs in exam preparation can enhance students’ learning.

Taken together, the findings of the current study suggest a role for SMPs in promoting language learning. The current study also contributes to the body of knowledge by providing evidence that students preferred to follow some proportion of social constructivism and connectivism theories, such as social interaction and the notion of the “more capable other”, in their preparation for exams and learning.

The pedagogical implications are for learners, stakeholders, educational policy makers and syllabus designers. For learners, these platforms could be a useful and encouraging environment to study, learn, discuss, collaborate with and expose to more input and information. The SMPs provide easy ways for learners to contact their instructors and find the information they are seeking. Students can create channels for each course to have an on-demand platform where they can share materials that are related to the exams. Available resources on these platforms are beneficial for learners in their future learning. The results showed that there is a positive effect of using SMPs in preparing for exams as they are platforms for support and the lowering of test anxiety. This study suggests using SMPs for educational purposes, namely as a medium for study.

Instructors, on the other hand, can activate SMPs, other Virtual Learning Environments such as Blackboard and educational apps such as Edmodo as a means of communication with their students specifically for test preparation purposes. Engaging students on these platforms can create 24/7 open channels for communication as it would be an easier way for instructors to follow up and update with their students. Moreover, whenever more than one instructor teaches the same course using SMPs to interact with students, it can help students draw a conclusion about the type of exam and the topics covered in the exam. Providing the learners with perfect answers for the tests after taking the test, may help learners to spot their mistakes and learn from them.

Instructors can also assign a module leader to the groups whom instructors can refer to if they need to provide certain information regarding tests or to deliver learners' questions from the learners to their instructors. The present results are insightful for using other available educational platforms such as Edmodo and Blackboard that could be monitored by instructors in order to present a suitable environment for a better education which enhances the process of learning and teaching. As asserted by Ref. [64]; monitoring students’ collaborative learning and discussions will help instructors to estimate and revise their materials through perceiving how students understand and master the intended knowledge and what parts are needed to be delivered in another way. Instructors would be able to create accounts in particular SMPs in order to offer extra materials and notes in relation to exams.

As for the educational policy makers, administrators may provide instructors with facilities that will help them to integrate such platforms in their courses. Making SMPs effective and efficient tools by the instructors would also help the students to use them for discussion, sharing information and exchanging ideas about their exams.

Decision makers can make use of the current study findings through determining ways to activate and make use of the facilities and affordances offered by those SMPs and other educational platforms. They can also educate instructors how to best invest in the SMPs that will enhance students’ learning and enable them to make use of the available structured environment learning in the university.

### 5.2 Recommendations for further investigations

This research has identified many areas in need of further investigation.

• The results of this study suggest that studying the use of SMPs in preparing for a specific language skill will be an interesting research area.
• The present study, for cultural reasons, included only female EFL learners. Studying the differences between male and female EFL students could be an open area of research and may contribute interesting findings to the literature of the field.
• A replication of this study with the same questionnaire is highly recommended as we need more confirmed results about the use of SMPs in test preparation, so that we can bridge the gap in this field.
• Different themes arose from the participants' answers regarding the use of SMPs in exam preparation, such as the ease of access to information, and speed of interaction with peers and instructors, all of which can be investigated in further research.
• There are still many variables such as learners' proficiency in relation to the use of SMPs in exam preparation. It would be interesting to see whether highly proficient learners use SMPs in exam preparation more than less proficient learners.

## Data availability statement

The authors do not have permission to share data.

## Ethical approvals

This research project was approved by the ethical committee at the Deanship of Scientific Research in King Faisal University, Saudi Arabia under the request number Ethics 52. Researchers obtained the informed consent of all participants.

## Funding

This work was funded and supported by the Deanship of Scientific Research, Vice Presidency for Graduate Studies and Scientific Research, King Faisal University, Saudi Arabia [Grant No. 4,577]

## CRediT authorship contribution statement

**Dr Abdullah A. Al Fraidan:** Conceptualization, Data curation, Formal analysis, Funding acquisition, Investigation, Methodology, Project administration, Resources, Supervision, Validation, Visualization, Writing – original draft, Writing – review & editing. **Ebtisam Mohammed Al-Harazi:** Data curation, Methodology, Writing – original draft.

## Declaration of competing interest

The authors declare that they have no known competing financial interests or personal relationships that could have appeared to influence the work reported in this paper.

## Footnotes

Appendix A Supplementary data to this article can be found online at https://doi.org/10.1016/j.heliyon.2023.e21320.
