# Supplementary material for: Using social media platforms to prepare for examinations post Covid-19: The case of saudi university EFL learners
Source: Heliyon. 2023 Oct 21;9(11):e21320. doi: 10.1016/j.heliyon.2023.e21320 (PMC10598519; doi:10.1016/j.heliyon.2023.e21320)
Supplement: Multimedia component 1 [file mmc1.docx]

**Appendices**

**Appendix 1: The Questionnaire (in English)**

The objective of this study is to determine whether EFL Saudi learners at King Faisal University in Al-Ahsa continued using Social Media Platforms (SMPs) to prepare for their exams after Covid-19.

**Part One: Primary information:**

Please answer the following before you continue answering the questionnaire items.

Put a tick to indicate correct information:

General introductory items:

A. For each statement, please choose one item that indicates your answer.

1. Gender

o Male

o Female

2. Type of enrollment

o Regular

o Distance Education

**Part two: Please answer the following questions:**

          2.A Do you still use Social Media platforms to prepare for exams?

o Yes

o No

2.B Which of the following Social Media platforms you use for exam preparation (Choose all the items that apply)

o I don’t use any for exam preparation

o WhatsApp

o Twitter

o Snapchat

o Instagram

o Facebook

o Telegram

o YouTube

o Others: Please Specify …………………

2.C When I use SMPs to prepare for exams, I interact with (Choose the items that apply)

o Friends

o Classmates

o Teachers / Instructors

o Others: Please Specify……………

Part Three:

3.A Please indicate how Social Media Platforms can help you in exam preparation.

Please put ( ) in the box that indicates your answer .

| (1)  Strongly Disagree | (2)  Disagree | (3)  Neutral | (4)  Agree | (5)  Strongly Agree |
| --- | --- | --- | --- | --- |

| S.N. | The statement | 1 | 2 | 3 | 4 | 5 |
| --- | --- | --- | --- | --- | --- | --- |
| 1. | I still use SMPs to discuss the topics covered in the exam with my classmates. |  |  |  |  |  |
| 2. | I still use SMPs to ask my classmates about any doubt I have regarding the topics covered in the exam. |  |  |  |  |  |
| 3. | I still use SMPs to share previous exams papers with my classmates. |  |  |  |  |  |
| 4. | I still use SMPs to revise the topics covered in the exam after studying them individually. |  |  |  |  |  |
| 5. | I still use SMPs to contact my instructors to ask for clarifications. |  |  |  |  |  |
| 6. | I still use SMPs to share expected exam questions with my friends. |  |  |  |  |  |
| 7. | I stil use SMPs in every exam preparation. |  |  |  |  |  |
| 8. | Having a group in SMPs for each course is very useful to study for exams. |  |  |  |  |  |

3.B When preparing for exams, how useful is using Social Media Platforms.

Please put a tick in the box that indicate your answer.

| (1)  Completely Disagree | (2)  Disagree | (3)  Neutral | (4)  Agree | (5)  Completely Agree |
| --- | --- | --- | --- | --- |

| S.N. | The Statement | 1 | 2 | 3 | 4 | 5 |
| --- | --- | --- | --- | --- | --- | --- |
| 1. | Using SMPs to prepare for exams help me increase my grades. |  |  |  |  |  |
| 2. | I find SMPs helpful learning environment to study. |  |  |  |  |  |
| 3. | I find many resources and topics of discussion that are helpful when using SMPs. |  |  |  |  |  |
| 4. | Debating about ideas and exchanging opinions with others using SMPs help me understand the topics covered in the exam better. |  |  |  |  |  |
| 5. | I express my opinions and thoughts more freely with SMPs than in face-to-face discussions with my classmates. |  |  |  |  |  |
| 6. | I can ask any question I have freely with SMPs than in face-to-face discussions with my instructors. |  |  |  |  |  |
| 7. | SMPs help me benefit from excellent students. |  |  |  |  |  |
| 8. | The most useful part in SMPs is that I can study anywhere and anytime. |  |  |  |  |  |
| 9. | SMPs motivate me to study more compared to traditional methods. |  |  |  |  |  |
| 10. | When I use SMPs, I can get a lot of necessary information in a short period of time. |  |  |  |  |  |
| 11. | I have noticed that my capability to recall information becomes better when I study using SMPs. |  |  |  |  |  |
| 12. | I believe that SMPs groups distract me from studying. |  |  |  |  |  |
| 13. | I believe that studying with the help of SMPs can be time-consuming. |  |  |  |  |  |

**Part Four:**

Please choose how often you use each type of the following Social Media Platforms to prepare for exams.

Please put (   ) in the box that indicate your answer.

| (1)  Never | (2)  Rarely | (3)  Sometimes | (4)  Often | (5)  Frequently |
| --- | --- | --- | --- | --- |

|  | Social Media Platform | 1 | 2 | 3 | 4 | 5 |
| --- | --- | --- | --- | --- | --- | --- |
| 1. | Facebook |  |  |  |  |  |
| 2. | Twitter |  |  |  |  |  |
| 3. | YouTube |  |  |  |  |  |
| 4. | WhatsApp |  |  |  |  |  |
| 5. | Telegram |  |  |  |  |  |
| 6. | BBM |  |  |  |  |  |
| 7. | Snapchat |  |  |  |  |  |
| 8. | Instagram |  |  |  |  |  |
| 9. | Others: please, write them in the blank ………………………………. |  |  |  |  |  |

**Part Five: Open-ended Questions**

5.A ) Does using SMPs have a negative or positive effect on your learning which affects your GPA? Why?

………………………………………………………………………………………………………………………………………………………………………………………………………………………………………………………………………………………………………………………………………………………………………………………………………………………………………………………………………………………………………………………………………………………………………………………………………………………………………………………………………………………………

5.B) What are the features that make you prefer using SMPs in exam preparation?

………………………………………………………………………………………………………………………………………………………………………………………………………………………………………………………………………………………………………………………………………………………………………………………………………………………………………………………………………………………………………………………………………………………………………………………………………………………………………………………………………………………………………………………………………………………………………………………………………………………………………………………………………………………………………………………………………………………………………………………………………………………………………………………………………………………………………………………………………………………………………………………………

5.C) Please add any additional answers regarding how you use SMPs in exam preparation.

………………………………………………………………………………………………………………………………………………………………………………………………………………………………………………………………………………………………………………………………………………………………………………………………………………………………………………………………………………………………………………………………………………………………………………………………………………………………………………………………………………………………………………………………………………………………………………………………………………………………………………………………………………………………………………………………………………………………………………………………

Thank you for your cooperation.

Ebtisam Alharazi

**Appendix 2: The Reviewers of The Questionnaire**

|  | 1. Dr. Anwer Al-Zahrani |
| --- | --- |
|  | 2 .Dr. Talal Alghizzi |
|  | 3. Dr. Miriam Al Kubaidi |
